# Determining the joint effect of obesity and diabetes on functional disability at 3-months and on all-cause mortality at 1-year following an ischemic stroke

**DOI:** 10.1186/s12902-018-0255-1

**Published:** 2018-06-18

**Authors:** Colleen Bauza, Sharon D. Yeatts, Keith Borg, Gayenell Magwood, Renee’ H. Martin, Anbesaw Selassie, Marvella E. Ford

**Affiliations:** 1 Department of Public Health Sciences, Medical University of South Carolina, Charleston, SC USA; 2 Department of Health Informatics, Johns Hopkins All Children’s Hospital, 601 5th Street South, Suite 707, St. Petersburg, FL 33701 USA; 3 Department of Emergency Medicine, Medical University of South Carolina, Charleston, SC USA; 4 Department of Nursing, Medical University of South Carolina, Charleston, SC USA

**Keywords:** Joint effect, Obesity, Diabetes, All-cause mortality, Functional disability

## Abstract

**Background:**

Obesity and diabetes mellitus, or diabetes, are independently associated with post-ischemic stroke outcomes (e.g.*,* functional disability and all-cause mortality). Although obesity and diabetes are also associated with post-ischemic stroke outcomes, the joint effect of obesity and diabetes on these post-ischemic stroke outcomes has not been explored previously. The purpose of the current study was to explore whether the effect of obesity on post-ischemic stroke outcomes differed by diabetes status in a cohort of acute ischemic stroke subjects with at least a moderate stroke severity.

**Methods:**

Data from the Interventional Management of Stroke (IMS) III clinical trial was analyzed for this post-hoc analysis. A total of 656 subjects were enrolled in IMS III and were followed for one year. The joint effects of obesity and diabetes on functional disability at 3-months and all-cause mortality at 1-year were examined.

**Results:**

Of 645 subjects with complete obesity and diabetes information, few were obese (25.74%) or had diabetes (22.64%). Obese subjects with diabetes and non-obese subjects without diabetes had similar odds of functional disability at 3-months following an ischemic stroke (adjusted common odds ratio, 1.038, 95% CI: 0.631, 1.706). For all-cause mortality at 1-year following an ischemic stroke, obese subjects with diabetes had a similar hazard compared with non-obese subjects without diabetes (adjusted hazard ratio, 1.005, 95% CI: 0.559, 1.808). There was insufficient evidence to declare a joint effect between obesity and diabetes on either the multiplicative scale or the additive scale for both outcomes.

**Conclusions:**

In this post-hoc analysis of data from the IMS III clinical trial of acute ischemic stroke patients with at least a moderate stroke severity, there was not sufficient evidence to determine that the effect of obesity differed by diabetes status on post-ischemic stroke outcomes. Additionally, there was not sufficient evidence to determine that either factor was independently associated with all-cause mortality. Future studies could differentiate between metabolically healthy and metabolically unhealthy patients within BMI categories to determine if the effect of obesity on post-stroke outcomes differs by diabetes status.

## Background

Obesity and diabetes mellitus, or diabetes, are not only highly prevalent in both the general US and international populations [1–4], but these factors are also prevalent among individuals who have been diagnosed with an ischemic stroke [5]. It is estimated that between 18 and 44% of individuals who previously had an ischemic stroke are obese, and between 25 and 45% of individuals who previously had an ischemic stroke have diabetes [5].

Stroke is a leading cause of long-term disability and death [6]. As a result, it is important to target modifiable factors in order to reduce the burden of these post stroke outcomes. Obesity and diabetes are independently associated with functional disability [7–12] and all-cause mortality [7, 13–26] following an ischemic stroke. Although obesity is a modifiable risk factor for ischemic stroke [27, 28], the reported effects of obesity on post-stroke outcomes of functional disability and of all-cause mortality have been conflicting. Whereas studies of the general population have found that increasing body mass concurrently increases the risk of functional disability [29] and of all-cause mortality [30, 31], a number of observational studies in a stroke population have reported that obesity is associated with a decreased risk of functional disability [7–9] and all-cause mortality [7, 13–18, 20]; this apparent discrepancy is referred to as the obesity paradox. As a result of these findings, the American Heart Association and American Stroke Association recommend all individuals who are diagnosed with an ischemic stroke be screened for obesity [5]. However, these agencies do not recommend weight reduction for overweight or obese individuals due to the null results of the Look Action for Health in Diabetes trial, a clinical trial that randomized overweight and obese individuals with type 2 diabetes to intensive behavioral intervention or usual care to compare the risk of vascular events (e.g.*,* stroke, myocardial infarction, or vascular death) [5, 32]. Despite evidence supporting the obesity paradox in the stroke literature as well as in the literature of other chronic diseases such as myocardial infarction, heart failure, and renal disease [33, 34], several investigators have questioned the validity of studies supporting the ‘obesity paradox,’ citing methodological issues (e.g.*,* the measurement of obesity, duration of obesity, treatment and/or selection bias due to the study population) or residual confounding as potential explanations [33–36]. In contrast to the conflicting reported effects of obesity on functional disability and all-cause mortality following a stroke, prior studies have established that diabetes is consistently associated with higher rates of functional disability [10–12] and higher risk of all-cause mortality [21–26] following a stroke.

Although obesity is a strong predictor of diabetes [37, 38], it is unknown whether diabetes modifies the inflammatory effects of obesity on functional disability or on all-cause mortality after an ischemic stroke. Research has recently supported the heterogeneity of the metabolic profile among obese individuals [39, 40], which suggests that the effect of obesity on functional disability and all-cause mortality following an ischemic stroke may differ according to diabetes status. The primary objective of this post-hoc analysis was to explore whether the effect of obesity on functional disability and all-cause mortality following an ischemic stroke differed by diabetes status.

## Methods

### Study population

This present study used data from the Interventional Management of Stroke (IMS) III clinical trial (IMS III, ClinicalTrials.gov number NCT00359424) [41]. Details of the scientific rationale, eligibility requirements, and baseline characteristics of the IMS III subjects have been published elsewhere [41, 42]. Briefly, the objective of the IMS III trial was to determine if subjects treated with a combined approach of intravenous recombinant tissue plasminogen activator (IV rt-PA) and endovascular therapy were more likely to have a better functional outcome than subjects treated with standard IV rt-PA alone [41, 42]. Eligibility was restricted to subjects between 18 and 80 years old, initiated with IV rt-PA within 3 h of ischemic stroke onset, and with a moderate-to-severe ischemic stroke, defined by a baseline National Institutes of Health Stroke Scale (NIHSS) score of at least 8 [41, 42]. Prior to enrollment, written informed consent was obtained from subjects (or a legal representative) [41, 42]. Subjects were followed for one year after onset of the ischemic stroke [41]. The Data and Safety Monitoring Board recommended the trial to stop in April 2012, after 656 subjects were randomized, due to crossing the pre-specified boundary for futility [42]. Specifically, the trial failed to show a benefit in functional outcome for the combined approach of IV rt-PA and endovascular therapy compared with standard IV rt-PA alone [42].

### Exposures of interest

Obesity and diabetes are the exposures of interest for this study. Based on source documentation and the IMS III Case Report Form Guidelines, obesity (yes, no) and diabetes (yes, no) were collected at the baseline visit. No further information was included in the Case Report Form Guidelines regarding the source for identifying this information (i.e. medical record documentation, patient reported history of disease, medically documented history of disease, lab test).

### Outcomes

The outcomes of interest for this study include functional disability at 3-months and all-cause mortality at 1-year following an ischemic stroke. Functional disability was measured using the modified Rankin scale (mRS), a 7-point ordinal scale that measures a subject’s degree of functional disability in daily activities after suffering from a stroke [43]. The mRS ranges from 0 to 6, with higher scores indicating greater functional disability [43]. For the current study, the full scale of the mRS was analyzed in order to incorporate response information from all categories. The mRS categories of 5 and 6 were collapsed into a single category based on the opinions of stroke subjects who indicated that being severely disabled (i.e.*,* category 5) is just as bad as or worse than death (i.e.*,* category 6) [44]. All-cause mortality at 1-year was defined as death due to any cause.

### Baseline data

A number of potential confounders were considered in the modeling approach on the basis of prognostic value or consistency within the literature [7–19, 21–26]. Multivariable models for each outcome were fit including pre-specified variables that were forced into the final model in addition to potential confounders, which are shown in Table 1.

**Table 1:** Variables and Definitions of Pre-Specified Variables and Potential Confounders for Analysis

Variables	Definition
**Pre-Specified Variables**	
Age^a,b^	≤ 65 years, > 65 years
Gender^a,b^	Male, Female
Race/ethnicity ^a,b^	White, Black/Other
Treatment assignment ^a,b^	IV rt-PA + Endovascular therapy, IV rt-PA
Baseline stroke severity ^a,b^	NIHSS < 20, NIHSS ≥20
Ischemic stroke sub-type ^a,b^	Large-artery atherosclerosis, Cardioembolic, Small-artery occlusion/Other/Unknown
**Potential Confounders**	
Baseline systolic blood ressure ^a,b^	< 140 mmHg, ≥ 140 mmHg
Baseline diastolic blood pressure ^a,b^	in mmHg
Baseline glucose ^a,b^	in mmol/L
History of previous stroke ^a,b^	Yes, No
History of atrial fibrillation ^a,b^	Yes, No
History of coronary artery disease ^a,b^	Yes, No
History of hypertension ^a,b^	Yes, No
Smoking status ^a,b^	Current smoker, Former/Never smoker
Alcohol use ^a,b^	Current drinker, Former/Never drinker

^a^Potential confounder for functional disability; ^b^Potential confounder for all-cause mortality

### Statistical analysis

All subjects were followed from the date of enrollment until the date of death, loss to follow-up, or the end of their 1-year follow-up, whichever occurred first. The relationship between functional disability at 3-months following an ischemic stroke and exposures of obesity and diabetes was modeled via proportional odds regression. A cross-product interaction term was used to derive adjusted common odds ratios (OR) and 95% confidence intervals (CI). The proportional odds assumption was assessed for all exposure variables and potential confounders using the Score test. The relationship between all-cause mortality at 1-year following an ischemic stroke and exposures of obesity and diabetes was modeled via Cox proportional hazards regression. A cross-product interaction term was used to derive adjusted hazard ratios (HR) and 95% CIs. The proportional hazards assumption was verified for all exposure variables and potential confounders using Schoenfeld residuals and time-dependent covariates [45]. For both models, multicollinearity between covariates was assessed by calculating individual variance inflation factors for each of the exposure variables and the potential confounders.

The joint effect of obesity and diabetes was examined on both the multiplicative and additive scales. The likelihood ratio test of the cross-product interaction term was used to determine the significance of the joint effect on the multiplicative scale. The joint effect on the additive scale, or the biologic interaction, was evaluated by two indices: the relative excess risk because of the interaction (RERI); and the attributable proportion because of the interaction (AP) [46]. RERI is an estimate of the excess risk attributable to the joint effect of obesity and diabetes and AP is defined as the proportion of risk attributable to the joint effect of obesity and diabetes [46]. These indices, along with their 95% CIs, were constructed using the approach of Li and Chambless [47]. A value of 0 indicates that there is no biologic interaction present [47, 48].

All statistical tests were two-sided and used an alpha-level of 0.05 with the exception of the joint effect on the multiplicative scale. For the joint effect on the multiplicative scale, statistical significance was defined at an alpha-level of 0.10, rather than 0.05, because clinical trials are not designed to detect a joint effect, only a main effect [49]. Statistical analyses were conducted using SAS software package version 9.4 (SAS Institute, Cary, NC). Institutional Review Board approval for this analysis was obtained from the Medical University of South Carolina (Pro00063231).

## Results

### Baseline characteristics of the IMS III study sample

Of the 656 IMS III subjects who were enrolled and randomized, obesity or diabetes information was not available for 11 (1.68%) subjects. Baseline characteristics according to obesity and diabetes information are shown in Table 2. Among these 645 subjects with complete obesity and diabetes information, few subjects were obese (25.74%) or had diabetes (22.64%). The majority of subjects were older than 65 years (58.45%), male (51.78%), white (84.50%), had a history of hypertension (74.73%), and were former/never smokers (75.19%). Among subjects without diabetes, obese subjects were more likely to have the following characteristics: be younger than 65 years, female, have a history of hypertension, have a baseline systolic blood pressure of at least 140 mmHg, and have a higher baseline median glucose. Among subjects with diabetes, obese subjects were also more likely to be younger than 65 years and have a higher baseline median glucose but were more likely to be male, white, and have a baseline systolic blood pressure of at least 140 mmHg.

**Table 2:** Baseline Characteristics of IMS III Subjects and by Obesity Categories and Diabetes Status

Characteristic	All subjects	No Diabetes		Diabetes	
		Non-Obese	Obese	Non-Obese	Obese
	No. (%)	No. (%)	No. (%)	No. (%)	No. (%)
No. of subjects ^a^	645	398	101	81	65
Obese					
Yes	166 (25.74)				
No	479 (74.26)				
Diabetes					
Yes	146 (22.64)				
No	499 (77.36)				
Sociodemographic Characteristics					
Age					
> 65 years	377 (58.45)	230 (57.79)	48 (47.52)	64 (79.01)	35 (53.85)
Gender					
Male	334 (51.78)	222 (55.78)	37 (36.63)	37 (45.68)	38 (58.46)
Race/ethnicity					
White	545 (84.50)	339 (85.18)	87 (86.14)	61 (75.31)	58 (89.23)
Black/Other	100 (15.50)	59 (14.82)	14 (13.86)	20 (24.69)	7 (10.77)
Clinical Characteristics					
Qualifying stroke subtype					
Large vessel atherosclerosis	127 (19.69)	70 (17.59)	24 (23.76)	18 (22.22)	15 (23.08)
Cardioembolic	299 (46.36)	185 (46.48)	46 (45.54)	37 (45.68)	31 (47.69)
Small vessel disease/Other/Unknown	219 (33.95)	143 (35.93)	31 (30.69)	26 (32.10)	19 (29.23)
Baseline stroke severity					
Severe (NIHSS ≥20)	198 (30.99)	6.3 (5.6-7.5)	6.7 (5.9-7.4)	8.2 (6.2-10.0)	8.8 (6.9-12.5)
Baseline glucose (median, IQR)	6.7 (5.7-8.1)	6.3 (5.6-7.4)	6.7 (5.9-7.4)	8.2 (6.1-10.0)	8.8 (6.9-12.3)
Baseline systolic blood pressure					
≥ 140 mmHg	386 (60.60)	219 (55.73)	67 (67.68)	62 (77.50)	38 (58.46)
Baseline diastolic blood pressure (median, IQR)	81 (71-94)	80 (70-93)	85 (70.5-98)	85 (73-96)	80 (71-91)
Treatment assignment					
IV rt-PA+ Endovascular therapy	429 (66.51)	274 (68.84)	61 (60.40)	50 (61.73)	44 (67.69)
IV rt-PA	216 (33.49)	124 (31.16)	40 (39.60)	31 (38.27)	21 (32.31)
Risk Factors and Comorbidities					
Smoking status					
Current smoker	160 (24.81)	106 (26.63)	30 (29.70)	14 (17.28)	10 (15.38)
Alcohol use					
Current drinker	251 (38.91)	165 (41.46)	43 (42.57)	21 (25.93)	22 (33.85)
History of a previous stroke					
Yes	86 (13.33)	46 (11.56)	9 (8.91)	21 (25.93)	10 (15.38)
History of hypertension					
Yes	482 (74.73)	269 (67.59)	79 (78.22)	72 (88.89)	62 (95.38)
History of coronary artery disease					
Yes	170 (26.36)	90 (22.61)	22 (21.78)	37 (45.68)	21 (32.31)
History of atrial fibrillation					
Yes	192 (29.77)	120 (30.15)	30 (29.70)	22 (27.16)	20 (30.77)

^a^11 subjects were excluded due to missing obesity or diabetes information

*IMS III* Interventional Management of Stroke III, *NIHSS* National Institutes of Health Stroke Scale, *IV rt-PA* Intravenous recombinant tissue plasminogen activator, *IQR* Interquartile range

### Joint effect of obesity and diabetes on functional disability at 3-months

**Fig. 1:**
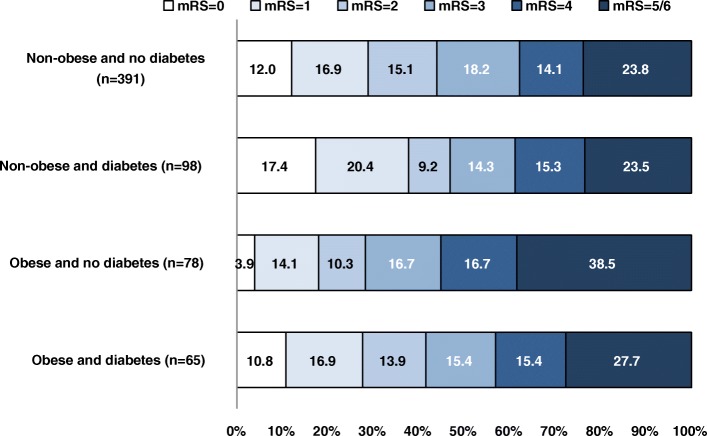
Distribution of modified Rankin Scale scores at 3-months following an ischemic stroke. Distribution of scores on the modified Rankin Scale at 3-months following an ischemic stroke according to obesity and diabetes in 632 IMS III subjects. mRS – modified Rankin Scale; IMS III – Interventional Management of Stroke III

The adjusted joint effect of obesity and diabetes on functional disability at 3-months following an ischemic stroke is shown in Table 3. Obese subjects with diabetes had similar odds of functional disability at 3-months compared with the reference group (common OR, 1.038, 95% CI: 0.631, 1.706). Similarly, there was not sufficient evidence to declare a joint effect between obesity and diabetes on either the multiplicative scale (*P*_interaction_, 0.6746) or the additive scale (RERI, 0.078, 95% CI: -0.260, 0.416; AP = 0.075, 95% CI: -0.169, 0.319). To further illustrate the distribution of functional disability at 3-months following an ischemic stroke, the mRS scores according to obesity and diabetes are displayed using Grotta bars in Fig. 1.

**Table 3:** Adjusted Common ORs for Functional Disability at 3-months in Relation to Obesity and Diabetes

Functional Disability at 3-months following the ischemic stroke	Obesity Categories	
	Non-obese	Obese
	OR (95% CI)	OR (95% CI)
Diabetes		
No	1.00	0.704 (0.466, 1.063)
Yes	1.256 (0.780, 2.020)	1.038 (0.631, 1.706)
Joint effect (additive):		
RERI (95% CI) AP (95% CI)		0.078 (-0.260, 0.416) 0.075 (-0.169, 0.319)
Joint effect on the multiplicative scale: *p*-value		*P*=0.6746

ORs are adjusted for age, gender, race/ethnicity, ischemic stroke sub-type, baseline stroke severity, baseline glucose, treatment assignment, smoking status, alcohol use, history of previous stroke, history of hypertension, and history of coronary artery disease

*RERI* Relative excess risk due to interaction, *AP* Attributable proportion due to interaction

### Main effects of obesity and diabetes on functional disability at 3-months

There was insufficient evidence to demonstrate that obesity was associated with increased odds of functional disability at 3-months following an ischemic stroke (Table 4, common OR: 0.740, 95% CI: 0.524, 1.044), after adjusting for diabetes and other factors. Similarly, there was also not sufficient evidence to determine that diabetes was not associated with increased odds of functional disability at 3-months following an ischemic stroke (common OR, 1.339, 95% CI: 0.924, 1.941), after adjusting for obesity and other factors.

**Table 4:** Adjusted Effect Measures for Associations between Obesity, Diabetes and Outcomes of Interest

Outcome	
3-month functional disability^a^	OR (95% CI)
Obesity	0.740 (0.524, 1.044)
Diabetes	1.339 (0.924, 1.941)
All-cause mortality^b^	HR (95% CI)
Obesity	1.092 (0.744, 1.602)
Diabetes	0.983 (0.638, 1.514)

^a^ORs are adjusted for age, gender, race/ethnicity, ischemic stroke sub-type, baseline stroke severity, baseline glucose, treatment assignment, smoking status, alcohol use, history of previous stroke, history of hypertension, and history of coronary artery disease

^b^HRs are adjusted for age, gender, race/ethnicity, ischemic stroke sub-type, baseline stroke severity, baseline glucose, baseline diastolic blood pressure, treatment assignment, smoking status, alcohol use, history of hypertension, and history of previous stroke

### Joint effect of obesity and diabetes on all-cause mortality at 1-year

The adjusted joint effects of obesity and diabetes on all-cause mortality at 1-year following an ischemic stroke are shown in Table 5. Obese subjects with diabetes had a similar hazard of all-cause mortality at 1-year following an ischemic stroke compared with the reference group (HR, 1.005, 95% CI: 0.559, 1.808). Furthermore, there was not sufficient evidence to declare a joint effect between obesity and diabetes on either the multiplicative scale (*P*_interaction_, 0.5311) or the additive scale (RERI, − 0.257, 95% CI: -0.842, 0.327; AP = − 0.256, 95% CI: -0.557, 0.045).

**Table 5:** Adjusted HRs for All-Cause Mortality at 1-year in Relation to Obesity and Diabetes

All-Cause Mortality at 1-year	Obesity Categories			
	Non-obese		Obese	
	Deaths/total	HR (95% CI)	Deaths/total	HR (95% CI)
Diabetes				
No	85/398	1.00	24/101	1.198 (0.743, 1.932)
Yes	27/81	1.064 (0.642, 1.761)	17/65	1.005 (0.559, 1.808)
Joint effect (additive):				
RERI (95% CI) AP (95% CI)				-0.257 (-0.842, 0.327) -0.256 (-0.557, 0.045)
Joint effect on the multiplicative scale: *p*-value				*P*=0.5311

HRs are adjusted for age, gender, race/ethnicity, ischemic stroke sub-type, baseline stroke severity, baseline glucose, baseline diastolic blood pressure, treatment assignment, smoking status, alcohol use, history of hypertension, and history of previous stroke

*RERI* Relative excess risk due to interaction, *AP* Attributable proportion due to interaction

### Main effects of obesity and diabetes on all-cause mortality at 1-year

There was insufficient evidence to demonstrate that obesity was associated with an increased hazard of all-cause mortality at 1-year following an ischemic stroke (Table 4, HR, 1.092, 95% CI: 0.744, 1.602), after adjusting for diabetes and other factors. Similarly, there was also not sufficient evidence to determine that diabetes was not associated with an increased hazard of all-cause mortality at 1-year following an ischemic stroke (HR, 0.983, 95% CI: 0.638, 1.514), after adjusting for obesity and other factors.

## Discussion

The purpose of this post-hoc analysis of data from the IMS III clinical trial of acute ischemic stroke patients with at least a moderate stroke severity was to explore the presence of a joint effect of obesity and diabetes on functional disability and on all-cause mortality following an ischemic stroke. Overall, there was not sufficient evidence to determine that the effect of obesity differed by diabetes status on functional disability at 3-months, or on all-cause mortality at 1-year, following an ischemic stroke on either the multiplicative scale or the additive scale. In addition, although obesity [7, 13–19] and diabetes [21–26] have been previously shown to be independently associated with all-cause mortality following a stroke, there was not sufficient evidence to determine that each factor was independently associated with all-cause mortality after adjusting for potential confounders in this cohort of acute ischemic stroke patients with at least a moderate stroke severity. In contrast, the point estimates for the independent associations between each factor and functional disability at 3-months following an ischemic stroke were consistent with the findings from the literature [7–12].

In comparison to some of the studies that cite the obesity paradox on post-stroke outcomes, there are several potential reasons for the discrepant results in the present study. First, the population only consisted of acute ischemic stroke subjects [33, 35, 50]. Some of the results from this study are consistent with several other studies that included only ischemic stroke subjects whereas the majority of the studies that support the obesity paradox included different patient populations (i.e.*,* only hemorrhagic [20], only ischemic [8, 9, 11–14, 16, 50, 51], stroke or TIA [7, 17], or both ischemic and hemorrhagic strokes [10, 15, 18, 19]). It is important to point out these differences in the study population because the pathogenesis of ischemic stroke is markedly different from that of hemorrhagic stroke, thus the effect of obesity on post-stroke outcomes may not be the same [52]. However, results of this study were similar to several other studies that only included recent ischemic stroke subjects [8, 9, 51]. Second, the outcomes of interest in studies that support the association between obesity and a decreased risk of all-cause mortality post-stroke were assessed at widely varying periods ranging from a week to 10 years [33, 35, 50]. However, the studies that had time points similar to the time points of acute stroke trials (IMS III, for example) determined that there was no functional or survival benefit for obese subjects [8, 50, 53]. Third, the inclusion of important prognostic factors, such as stroke severity and smoking use, as potential confounders differed across studies [33, 35, 50]. It is critical to account for these important confounders to reduce residual confounding, however many of the studies that assessed these associations did not account for these confounding variables. Lastly, the measure of obesity is nearly always body mass index (BMI). Although BMI is the most commonly used diagnostic tool for obesity in clinical practice [5, 54], BMI is unable to differentiate between body fat percentage and lean mass which leads to misclassification [55] nor does it tell the distribution of body fat. Rather than using BMI to measure obesity, it is critical to determine alternative diagnostic tools capable of differentiating risk of poor clinical outcomes following an ischemic stroke such as waist circumference or waist-to-hip ratio [33, 56].

The present study has a number of limitations that could influence the interpretation of the study results. Due to the restrictive criteria of the IMS III clinical trial, the results of the present study may not be generalizable to all acute ischemic stroke patients. For example, patients were excluded if they had mild stroke severity (NIHSS < 8). The generalizability of the results of this study is therefore limited to ischemic stroke patients with at least a moderate stroke severity who met all of the study eligibility criteria. Thus, the potential for selection bias cannot be excluded. Future research could be performed to determine if the results demonstrated among a cohort of acute ischemic stroke subjects with at least a moderate stroke severity would be similar to the results among a cohort of acute ischemic stroke subjects.

Additional identified limitations are associated with the measurement of the exposures of interest. Results of this study were limited in the interpretability of the results partially due to how obesity and diabetes information were captured (i.e.*,* binary summary measures). There may be measurement error based on the how obesity and diabetes information was ascertained. Specifically, no further definition of these variables was provided in the IMS III Case Report Form Guidelines. Therefore, we were not able to accurately define obesity or diabetes based on their BMI or fasting blood glucose levels, respectively. Although these measures are based on high-quality data, the degree of obesity or diabetes could not be determined at baseline. Thus, the potential for measurement bias cannot be excluded. Future studies could capture multiple measures of obesity, specifically BMI, waist circumference, and/or waist-to-hip ratio, rather than a summary indicator for obesity and/or utilize the World Health Organization’s public health action points [57] to further define subjects’ degree of obesity. These alternative measures would allow for greater interpretability. Additionally, the exposures of interest are only snapshots of subjects’ history of obesity and/or diabetes. As a result, it was not possible to determine the cumulative effect, or allostatic load, of either exposure of interest. Future studies could collect information on subjects’ weight histories in addition to the duration of diabetes to accurately determine whether the effect of obesity on post-ischemic stroke outcomes differs by diabetes status.

Additionally, IMS III was not designed to answer the research questions of the present study. Examining joint effects, or interactions, is challenging because tests for interactions are typically underpowered [58]. Despite these limitations and the confines of statistical power, this study was able to demonstrate the joint effect of obesity and diabetes on functional disability and on all-cause mortality following an ischemic stroke is insignificant. Although other analytical strategies were applied to offset these problems, it is imperative to strive for sufficient power to examine the potential joint effect of obesity and diabetes on clinical outcomes following an ischemic stroke. Thus, it is critical to utilize a national or international ischemic stroke registry that would provide sufficient resources and power for future studies to address these research questions.

Despite some limitations, the present study includes several notable strengths. First, this is the first study to explore the potential multiplicative and additive joint effects of obesity and diabetes on functional disability and all-cause mortality following an ischemic stroke. Results of this research provide evidence for generating hypotheses for future studies investigating how obesity and diabetes could potentially interact with one another to affect the clinical outcomes following an ischemic stroke. Second, the rigorous data collection of the IMS III trial reduced information bias. Rather than relying on subjects self-reporting their medical history, the use of source documentation to verify sociodemographic characteristics, clinical characteristics, and risk factors and comorbidities prevented bias that may have resulted from self-reporting. Third, IMS III investigators followed strict study procedures, which minimized the potential bias from incorrect documentation of the trial’s outcomes.

## Conclusions

Overall, it is important to continue to study joint effects of these common modifiable factors to identify susceptible subgroups of individuals that would potentially benefit from effective interventions targeted at reducing the burden of functional disability and all-cause mortality [58]. This topic is of high public health priority. Obesity and diabetes are not only highly prevalent in both the general US and international populations [1, 3, 4, 59], but they are also prevalent among individuals who have been diagnosed with a stroke [5]. It is estimated that between 18 and 44% of individuals who previously had an ischemic stroke are obese, and between 25 and 45% of individuals who previously had an ischemic stroke have diabetes [5]. Recent research has supported the heterogeneity of the metabolic profile among obese individuals [39, 40]. Overall, the underlying mechanisms by which obesity and diabetes may interact to affect functional disability or all-cause mortality following an ischemic stroke remain unclear. Thus, future studies should differentiate between metabolically healthy and metabolically unhealthy patients within BMI categories (or other diagnostic tools for obesity) to determine if the effect of obesity on post-ischemic stroke outcomes differs by diabetes (or some other metabolic health measure).

## Abbreviations

AP: The attributable proportion because of the interaction
CI: Confidence interval
HR: Hazard ratio
IMS III: Interventional Management of Stroke III
IV rt-PA: Intravenous recombinant tissue plasminogen activator
mRS: Modified Rankin Scale
NIHSS: National Institutes of Health Stroke Scale
OR: Odds ratio
RERI: The relative excess risk because of the interaction
